# Predicting Students’ Interest in Aging-Related Careers: Suggestions for Pedagogical Intervention

**DOI:** 10.1093/geroni/igab046.2794

**Published:** 2021-12-17

**Authors:** Chih-ling Liou

**Affiliations:** Kent State University, North Canton, Ohio, United States

## Abstract

College students often hold negative attitudes toward elders and rank this area of practice at the bottom of their future professional life; colleges and universities have an important role to play in changing attitudes and attracting more professionals to work with older adults. This study examined factors contributing to students’ attitudes toward older adults to provide suggestions for pedagogical intervention. Data was collected from 195 undergraduates participating in an online survey with questions on the quality of relationships with a grandparent and other nonfamilial older adults, previous experience and future interest in pursuing a career in an aging-related field, and The Fabroni Scale of Ageism (Fabroni et al., 2010). Path analyses using hierarchical multiple regression revealed that high quality relationships with older adults (i.e., both grandparents and nonfamilial elders) was associated with less negative attitudes and more interest in pursuing a future career in age-related jobs/internships. Although both types of relationship quality were significant in the model (p<.05), path coefficients demonstrated that relationships with nonfamilial elders have a greater impact on participants’ attitudes (β= -.250, p=.001 versus β= -.146, p=.045). Previous working/internship experiences with older adults also predicted a greater willingness to pursue a future career in an aging-related field (β= .333, p<.001). Findings suggest that colleges could increase students’ interest in pursuing aging-related careers with multiple interventions, such as developing opportunities to interact and build relationships with older adults in the community, updating information on job opportunities, pay scales, and advancement opportunities, and providing more gerontological course or modules.

